# Experiences of children with obesity and their parents of participating in a physical activity on prescription intervention: a qualitative study

**DOI:** 10.3389/fped.2026.1831386

**Published:** 2026-05-14

**Authors:** Elvira Lange, Katarina Lauruschkus, Stefan Lundqvist, Karin Melin, Susanne Bernhardsson

**Affiliations:** 1 Unit of Physiotherapy, Department of Health and Rehabilitation, Institute of Neuroscience and Physiology, Sahlgrenska Academy, University of Gothenburg, Gothenburg, Sweden; 2 Research, Education, Development and Innovation Primary Health Care, Regionhälsan, Region Västra Götaland, Gothenburg, Sweden; 3 General Practice/Family Medicine, School of Public Health and Community Medicine, Institute of Medicine, Sahlgrenska Academy, University of Gothenburg, Gothenburg, Sweden; 4 Department of Health Sciences, Faculty of Medicine, Lund University, Lund, Sweden; 5 Faculty of Health Sciences, Kristianstad University, Kristianstad, Sweden; 6 Center for Physical Activity, Regionhälsan, Region Västra Götaland, Gothenburg, Sweden; 7 Department of Pediatric Neurology and Psychiatry, Sahlgrenska University Hospital, Region Västra Götaland, Gothenburg, Sweden; 8 Gillberg Neuropsychiatry Centre, Institute of Neuroscience and Physiology, Sahlgrenska Academy, University of Gothenburg, Gothenburg, Sweden

**Keywords:** children, family-centred, interview, obesity, parents, physical activity on prescription, qualitative

## Abstract

**Introduction:**

The prevalence of childhood overweight and obesity has increased substantially in recent decades. Physical activity is a key component in improving metabolic and overall health in children with obesity. The Swedish Physical Activity on Prescription (PAP) is an established method for promoting physical activity, yet knowledge regarding its applicability and acceptability in paediatric populations is scarce. This study explored children's and parents’ experiences of PAP, including perceived barriers, facilitators, and any changes in physical activity behaviour.

**Methods:**

Twenty-two children and parents were purposively recruited from a previous intervention study at paediatric and rehabilitation clinics in Region Västra Götaland, Sweden. Semi-structured, in-depth interviews were conducted at locations chosen by participants, audio-recorded, and transcribed verbatim. Data were analysed using inductive qualitative content analysis, including coding, categorisation, and development of an overarching theme. Researcher triangulation was applied throughout the analytical process to strengthen credibility.

**Results:**

The analysis resulted in the main theme *Physical activity on prescription spanning healthcare and everyday life,* supported by five categories, each with three to five sub-categories. The category *The clinical encounter* describes the overall experience of PAP as part of a larger whole; *Finding “your thing”* addresses activity selection as a process with several factors influencing the choice, and *Fitting activities into everyday life* concerns adoption of activities, engaging the family, and strategies to sustain activities. The category *Motivation through monitoring and* f*ollow-up* describes three different types of follow-up important for motivation: a digital diary, healthcare contacts, and wearing a accelerometer waistband. The last category, *Changes beyond physical activity,* captures experiences of development in both activity, behaviour, health and emotion.

**Discussion:**

The findings suggest that children with obesity and their parents experience PAP as a supportive, person-centred, intervention extending across healthcare, everyday life, and organised sports. The intervention was perceived as combining conversational guidance with shared responsibility, with engagement facilitated by fun, tailored activities aligned with family routines, while financial and practical barriers could limit participation. Overall, the findings suggest that individually adapted, family-inclusive PAP is a feasible and acceptable strategy in Swedish paediatric obesity care, with potential broader benefits for children's health and well-being.

## Background

Overweight and obesity among children have increased markedly over recent decades and now constitute a major public health problem. In Sweden, obesity affected an estimated 7.2% of 6–9 years old children in 2022 (girls 7.4%, boys 7.1%), with widening socioeconomic disparities (1, 2). Rates increased further during the COVID-19 pandemic, particularly among younger children and those living in socioeconomically disadvantaged areas (3, 4). Among older children and adolescents, the pandemic period was additionally characterised by reduced physical activity and increased screen time (5).

Physical inactivity is a key contributor to childhood obesity (6) and is important to address early, as obesity in childhood often persists into adulthood (7). Physical activity has well-documented effects on children's physical and mental health and mitigates risk factors for lifestyle-related diseases, including type 2 diabetes, which is now diagnosed at increasingly younger ages (8). In children with overweight or obesity, physical activity improves blood lipid profiles and insulin sensitivity (9, 10), with greater effects when combined with dietary modification (11). Physical activity also confers important benefits for cardiovascular and metabolic health (12).

Swedish guidelines recommend that children aged 6–17 years engage in at least 60 min of daily physical activity at moderate-to-vigorous intensity, including vigorous intensity and muscle-strengthening activities several times weekly (13, 14). For children with obesity, physical activity three times weekly at approximately 75% of maximal heart rate is recommended to enhance metabolic outcomes (15). Although studies are not entirely consistent, parents’ physical activity levels and parental support are associated with children's activity behaviours (16, 17), underscoring the importance of involving parents in interventions aimed at increasing physical activity in children.

Physical activity on prescription (PAP) is a healthcare-based method to promote physical activity among patients (18). The Swedish PAP method includes a person-centred consultation, an individualised written prescription for one or more physical activities, and structured follow-up. Activities may be performed individually or in groups, and in various settings (19, 20). Studies have shown that Swedish PAP can increase physical activity levels among adults, including those with overweight or obesity (19, 20). Qualitative studies highlight that tailored physical activity, including written prescriptions and structured follow-up, enhance motivation and adherence, emphasising the importance of individualised components for achieving intended behavioural outcomes (21, 22).

However, research on PAP for children and adolescents is limited, both regarding effectiveness and experiences of participation among children and their parents. Existing studies primarily involve evaluations of PAP for children with obesity (23–25) and one qualitative study exploring parents' experiences of a multicomponent intervention in which PAP constituted only one component (26). No previous research has explored experiences of participating in PAP from both child and parent perspectives. In an ongoing research project, our research group is currently evaluating the feasibility of PAP for children with obesity. Prior to broader implementation of the PAP method in paediatric healthcare, it is essential to understand patients' experiences and perceptions of the intervention and to identify prerequisites for successful implementation. The aim of this study was therefore to explore children's and parents' experiences of participating in a PAP intervention, focusing on: (1) their overall experiences; (2) perceived barriers and facilitators; and (3) perceived behavioural changes in children following PAP treatment, particularly regarding physical activity.

## Methods

### Study design

An exploratory qualitative design with an inductive approach was used to capture participants' perspectives. The study is part of a larger research project on the feasibility of PAP for children with obesity in paediatric healthcare (27) and was conducted and reported in accordance with the Consolidated criteria for reporting qualitative research checklist (28).

### Participants and setting

Participants were recruited from a previous intervention study, conducted at five paediatric clinics and three rehabilitation clinics in Gothenburg and surrounding municipalities, Sweden (24, 25). During the final visit of that study, a research assistant provided oral and written information about the current study and invited eligible participants to take part. A purposive sampling strategy was used, striving for maximum variation in terms of child and parent sex, age, type of clinical setting (paediatric or rehabilitation), and country of origin. Eligible participants included children aged 7–13 years who had participated in the previous study, initially with an ISO-BMI above 30, along with one of their parents, who were also required to have taken part in the previous study.

Thirty-eight children/parents were invited to participate in the study, of whom 22 (eight girls, three boys and six mothers, five fathers) accepted. Mean age of the children was 10.6 (range 8–13) years. Parents' mean age was 39.9 (31–50) years. Six of the participating parents were Swedish-born, one was from southeast Europe, and four were of non-European origin. Three parents reported primary school as their highest education level, six reported secondary school, and two reported university degrees. The families lived in and were recruited from paediatric clinics located in areas varying in socioeconomic status across the city and surrounding municipalities.

### Intervention

The PAP intervention was adapted to the target population based on barriers and facilitators identified in two prior studies (29, 30) as well as input from parents. Key adaptations included the use of visual aids, parental involvement, and cultural tailoring. Following the initial counselling session, the intervention was further individualised through collaboration between the child, parent, and healthcare professional. The four-month intervention was delivered individually by trained paediatric healthcare professionals via in-person visits and digital modalities. A project-specific mobile application enabled access to PA prescriptions, activity logging, and communication with providers, as well as push notifications related to activity goal attainment intended to support adherence. Walking, ball sports, and swimming were the most frequently reported activities.

In the previous intervention study, the children's progress was assessed at four measuring points using accelerometry, self-report questionnaires, and medical chart data (24, 25). An accelerometer was worn by both children and parents in an elastic waistband for 7 days at each measuring point, supplemented by an activity diary. Questionnaires were completed by child and parent either on paper at the follow-up appointment or directly in the mobile application. These study components are normally not part of the PAP intervention.

### Data collection

Data collection took place between September 2023 and April 2025, at an average of 7 (range 2.5–17) weeks after completion of the 12-month follow-up in the previous study. Data were collected in semi-structured, in-depth interviews. The first author contacted those who had consented to participate and scheduled the interviews. The participants chose the interview location; seven of the interviews were held at the paediatric clinic, two at the research unit's premises, and two in the participants' home. An interview guide and visual aids were developed. The visual aids were used during the interview to describe and clarify the PAP treatment and its components to the participants to help them recall what the intervention was about, and to facilitate discussion about performed activities (Supplementary File 1). The interview guide contained an open-ended introductory question, six key questions, and follow-up prompts (Supplementary file 2). All questions were first directed to the child and secondly to the parent. The guide was pilot-tested in two interviews that involved a child and a parent, and in one also an interpreter. Practical aspects assessed included the interview setting, use of visual aids, time allocation for interpretation, and strategies to ensure engagement of both child and parent with focus on creating a safe environment. The interviewer and principal investigator jointly evaluated the pilot interviews, and concluded that the semi-structured guide captured both child and parent perspectives, required no modifications, and was feasible for joint child-parent interviews. The interviewer was introduced as an independent researcher not involved in the intervention.

In two of the interviews, an interpreter was present, and in three interviews, siblings were present. In the remaining interviews, only the child, the parent, and the interviewer were present. Field notes were made after each interview to sum up key characteristics of the participant's story. Data collection was terminated after 11 interviews based on assessment of information power, taking into account the study's aim, sample specificity, quality of dialogue, and richness of the gathered material (31). Two pilot interviews were conducted in September 2023, and the remaining nine interviews between November 2023 and April 2025. As the pilot interviews did not differ substantially from the subsequent interviews, they were included in the study. Interviews lasted between 22 and 52 min (mean 32 min), were audio-recorded, and transcribed. Transcription was performed with the assistance of the artificial intelligence-based tool KB-Whisper, a Swedish-language adaptation of OpenAI's Whisper automatic speech recognition model. The transcripts were verified and corrected by two authors (EL, SB).

### Data analysis

Data were analysed inductively using qualitative content analysis, following the procedure described by Graneheim and Lundman (32, 33). Both manifest and latent content were analysed; the former is presented in categories and the latter is expressed in an overarching theme. Unit of analysis was the entire interview. To gain a sense of the whole, the audio files were listened to and the transcripts were read several times with the study's aim in mind. Meaning units with content related to the study aim were identified, condensed, and coded. The first transcript was coded independently by the first author (EL) and the last author (SB), who subsequently met to discuss and agree upon a common coding strategy. Thereafter they coded half of the remaining transcripts each and verified each other's coding in an iterative process that involved comparison and revision of codes and categories. The codes were organised into subcategories and categories. Organisation and labeling of subcategories and categories were continually checked and modified throughout the analytical process. Categories were compared for differences and similarities aiming to keep them internally as homogeneous as possible and externally as heterogeneous as possible (32). Up to this point in the analysis, codes and categories were close to the data and on a descriptive level (manifest content). The second author (KL) verified content conformity of codes and categories. In the last stage of the analysis, an overarching theme that could be discerned across the categories was developed from the underlying (latent) content. Coding and categorisation were done in NVivo, version 15.

### Researcher characteristics

The interviews were conducted by the first author, a female physiotherapist with a PhD and both training and prior experience in qualitative research. She had no prior relationship with the participants or involvement in the underlying study. The second author is a female physiotherapist with a PhD, also with training and prior experience in qualitative research. The third author is a male physiotherapist with a PhD, with some experience in qualitative research, and the fourth author is a female specialist nurse with a PhD, also with training and prior experience in qualitative research. The last author is a female physiotherapist, associate professor and principal investigator of the project, with training and substantial experience in qualitative research. Researcher tasks and contributions are specified under Declarations.

## Result

**Figure 1:**
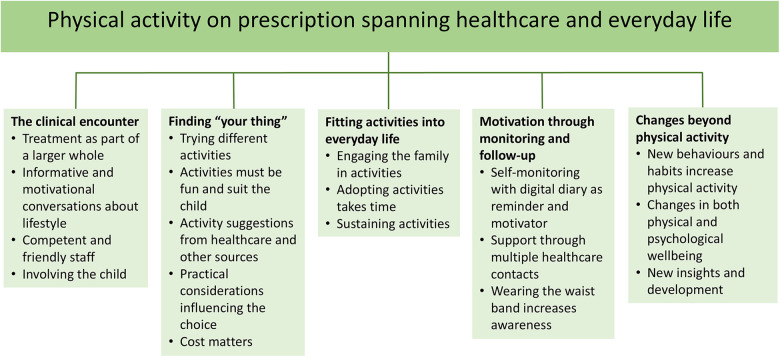
Overview of theme, categories and subcategories.

The analysis resulted in five categories, each with three to five sub-categories, and an overarching theme: *Physical activity on prescription spanning healthcare and everyday life* (Figure 1). The theme reflects how PAP was experienced as a method that extends across contexts—from healthcare, represented by the clinical encounter, through exploration, selection, adoption, and sustaining different activities and incorporating them into everyday life, to monitoring and follow-up that bridged the clinical and everyday domains. Participants described changes extending beyond physical activity, influencing the child's behaviour, health, and emotional wellbeing. Thus, PAP was experienced as an intervention embedded across, and dependent on, multiple layers of participants' lives.

Categories and subcategories are presented below, with supporting quotes in which child (C) and parent (P) are indicated.

### The clinical encounter

This category describes how the clinical encounter was experienced as an interaction characterised by guidance, encouragement, and shared responsibility for initiating and maintaining physical activity. The treatment was often experienced as a whole rather than as distinct parts, and participation involved conversations, guidance, and varying expectations of responsibility. The staff were generally perceived as competent, friendly, and encouraging, which contributed to a welcoming atmosphere. The child's involvement in both the treatment and discussions about lifestyle habits was described as meaningful, even when some aspects were experienced as challenging or not fully meeting expectations.

#### Treatment as part of a larger whole

Many participants had difficulty recalling specific components of the treatment. Instead, they described their experience as a whole, which could encompass both other medical services provided by the clinic and the purely study-related elements, particularly the measurement periods. A sense of satisfaction was expressed at having met various staff categories and been assessed. The PAP treatment was described by some as “just talk,” which for certain participants did not align with their expectations that healthcare providers would take a more active role in getting children physically active, while others acknowledged that the “work” was ultimately their own responsibility. At the same time, both children and parents expressed a broadly positive overall impression of PAP and described that everything had worked well.

Well, I thought so in the beginning, when we met them, that they would exercise with [the child]. They are the ones that will do the job. But they gave us advice, and it's us who did the job. (P11)

#### Informative and motivational conversations about lifestyle

The initial PAP consultation was described as an informative and motivational conversation covering feelings, activity habits, and the importance of physical activity—the latter being described as a facilitator for sustained motivation. Screen time was also addressed, and one parent described it as helpful that another adult, beyond the parents, discussed screen habits with the child. During follow-up conversations, advice was also provided about finding a balance in activity, e.g., not exercising too often or losing too much weight, which for some could be difficult to understand. One parent wished for more psychological support to better understand the child's personality and find tailored strategies.

Well, we started with talking and stuff. (C10)

About how important it is to move. (P10)

Yeah. And then I started to move more. Because they said it was good you know. (C10)

One family expressed a negative impression of the initial consultation, describing the extensive questioning as feeling like an interrogation and that visit as far too long.

It [the first consultation] was a lot of talk, it was well over two hours. I don't remember, it was a long time ago.//…// Honestly, the worst part was the length of the first visit. (P1)

We can say it took from three o'clock to five o'clock. (C1)

I felt like I was in a police interrogation. (P1)

#### Competent and friendly staff

The staff were perceived as competent and professional, and participants described a welcoming atmosphere in the clinical encounter. Several children described enjoying talking to the staff or getting to know them. The staff were found helpful and patient in offering suggestions, and participants expressed that receiving positive feedback felt good and motivating and facilitated the child's engagement. One dissenting view was a perception that the staff lacked knowledge about specific conditions related to certain activities.

I think the best was… It felt very welcoming and genuine as if people cared. (P10)

Yes. (C10) … and really wanted to help. (P10)

Well, for example, those who work here. They seem to know almost everything. (C6)

#### Involving the child

Children were described as being invited to take an active role in the clinical encounter, for example by being addressed directly and involved in activity choices. Initial discomfort was reported in one case, although this diminished as the child came to understand the purpose of the visits. At the same time, another parent described persistent difficulties engaging their child in the visits throughout the study.

It doesn't really help that the parents talk about it. The children need to be involved too. It's been very good. (P8)

### Finding “your thing”

This category reflects the children trying and discovering meaningful activities. Finding suitable activities was experienced as a process of trial-and-error, shaped by the child's preferences, practical considerations, and costs associated with the activity. They tried a number of different activities; some suggested by the healthcare professional as part of PAP, and some inspired by siblings, friends, or school. Benefits with the physical activity prescription, including discounts for activities or help bypassing admission queues, were perceived by parents as an important facilitator.

#### Trying different activities

Participants described trying several organised activities before settling on one they enjoyed. Sometimes an activity that they tried could lead them into another activity, considered more fun and replacing the initial activity; e.g., trying a play-oriented water activity but ultimately preferring competitive swimming. Children were typically positive and keen on trying something new but often lost interest quickly, sometimes causing frustration among parents. Activities initially selected but soon replaced by others included swimming and biking, while football was a popular activity that many children switched to. Other chosen activities included horseback riding, dance, judo, and jujutsu. Beyond organised activities, participants also described efforts to increase everyday activities such as biking, walking to and from school, as well as organising more family outings on weekends.

In the beginning he went to Capoeira classes. But then he stopped. And then we tried to motivate him to do something else. We suggested karate, table tennis, walks. But he never stuck to any of that. (P9)

#### Activities must be fun and suit the child

The importance of activities being fun and corresponding to the child's interest was underscored by both children and parents, as was the importance of the child being involved in selecting the activities. It was also important that the activities suited the child's preferences and personality; for example, a child uncomfortable with group activities appreciated training alone.

Yes, but you should see when she gets into the water. Sometimes she's a bit tired—after school and when it's late in the evening. A bit tired and not that motivated. But then it's like, “Oh yes, it’ll be fine,” you say, and then she gets there and jumps into the water and it's just… like that! (laughter) (P10)

Yes! The corners of her mouth are up by her ears. You can see that she really thinks it's fun. (P10)

Yes (C10).

#### Activity suggestions from healthcare and other sources

Participants described that available activities had been presented to them to various extents by the PAP provider. Some were satisfied with the suggestions, and were introduced to activities they did not know about. Some suggestions were for the whole family and some were only for the child. However, a lack of relevant suggestions was also described, particularly for activities that were fun and child-friendly. Communication between provider, child, and parent was not always considered optimal, leading to misunderstandings and activity suggestions that did not fit the child. Activity offerings were sometimes seen as limited in terms of time and location, for instance being offered just once per week at an inconvenient time. In addition to recommendations from the PAP provider, participants had also been exposed to activities through different channels in society, for example school or community-based arrangements. A school nurse was given as an example, who had provided suggestions to a child to start swimming. Another common source of activity suggestions were siblings or friends, which was described as an important facilitator to getting the child started.

They [the suggested activities] were pretty boring things. (P4)

Yes, it was stuff that like 40-year-old women do. Nothing wrong with that I mean… just not something I would want to do. I would have wanted more things that kids would like to do. Kids my age I mean. Like, admission to the swimming pool for a couple hours. With a friend or something. Or the swimming pool and something else. What else was there? (C4)

Well I think… well laser tag is a fantastic activity. Since they… wow what action in there, they give their everything! So more child-friendly activities. Instead it was sort of like—you can go to this gym class. (P4)

Yes. (C4)

#### Practical considerations influencing the choice

Several practical considerations influenced the choice of activities, many beyond the control of healthcare, functioning as facilitators or barriers for participating in organised activities or for adopting new everyday activities. One such consideration was the timing of the initial PAP consultation. A barrier described in this regard was that many sports activities vary by season, with sign-up at the beginning of the school semester—a system that could be additionally difficult to navigate without familiarity with Swedish conventions. Many activities were unavailable during summer, though the opposite was also described, such as a football club recruiting new members at school just before the summer. Cold or rainy weather posed a further seasonal barrier, limiting possibilities and motivation for outdoor activities.

All the activities took a summer break. (P8)

The timing of activities also mattered, for example activities colliding with other plans or a tight overall schedule. Another practical consideration was whether a sibling or friend was already involved in or able to join an activity, which was described as both facilitating and motivating by lowering the threshold to sign up. It could also be a coincidence, such as a parent's injury or a flat bicycle tire, that determined which activities were feasible at a given moment. Parental attitudes also mattered; one parent did not care much about going swimming with their child and instead they switched to taking longer walks together.

The kids like when you do different activities. It might just be the adults that don't want to be as active. (P11)

#### Cost matters

The cost of activities was described as a barrier, for participation or for signing up for an activity before knowing whether the child would like it. In some cases the prescription entitled participants to reduced fees, for example at gyms; a much appreciated possibility that was suggested should apply to more activities. The prescription could also facilitate access to an activity by allowing the child to bypass admission queues. Some participants did not, however, recall receiving a prescription, and one parent mentioned not using it because the discounted activity was not of interest.

During this whole time, have there been any barriers that you had to get passed? (Interviewer)

In the beginning I guess it was the economic aspects. But that has changed during the year I have to say. But there were a few things that were a bit of a barrier in the beginning. (P6)

In what way? Did it also affect availability of activities or was it something else? (Interviewer)

I guess it's the different choices, to try activities, and just in general to get started. And be able to continue. (P6)

### Fitting activities into everyday life

This category reflects how physical activity needs to be integrated in everyday family life. Participants described the importance of family engagement and involving siblings in activities. Adopting new activities was experienced as a gradual process that could take time, which could work both for and against sustained participation. Both children and parents recognised the importance of maintaining activities over time, and discussed facilitators and barriers for doing so, including the challenge of finding sufficient time and energy for physical activity.

#### Engaging the family in activities

Physical activity was described as something that takes place together with family members in everyday life. Parents and siblings take part by joining in, helping out, playing, and exercising together across a range of activities, including walks, outdoor play, gym visits, dancing, and cycling. In several cases the whole family was involved, with responsibility shared between different adults. Doing activities together was experienced as supportive and motivating, making participation in activities more feasible and enjoyable.

Sometimes her dad took us [to practice], sometimes mom, sometimes my bonus mom. It was fine. (C10)

So would you say that you've received the support you need for it to work? (Interviewer)

Yes (C10).

How was it for you [turns to the parent]? (Interviewer)

Well I live very far away. So … I can't be there as much as I would have wished. But I try to be there and to give encouragement, and I try to come along as much as I can. (P10)

#### Adopting activities takes time

Some participants described how it became easier to participate in activities after a while, as the child grew familiar with an activity. Starting something new could be challenging, but tended to become easier and more enjoyable once the activity was learned and the child accustomed. At the same time, an activity could also be experienced as exciting initially, but the child might lose interest after the initial phase.

It felt more as if you forced [the child] than for it to be fun. I think you can put it that way. Then it has sort of just developed. It's become fun to do things. (P5)

#### Sustaining activities

Both children and parents expressed a desire to be and remain active, with parents emphasising the importance of sustained physical activity for the child's health and well-being. Many participants also described enjoying their chosen activities. Different strategies to facilitate sustained activity over time were discussed, such as maintaining regular activity routines or structured weekly schedules, although these varied in effectiveness. Finding in an already busy everyday life was described as challenging, particularly if the child did not enjoy the activity, and in these situations, the healthcare provider could offer advice and support. Engaging children more in family life as they grew older was also mentioned as a strategy to manage household chores and free up time for physical activity.

Well barriers… weekdays it's a bit of a problem to find time. Both time and energy, I have to say. (P6)

### Motivation through monitoring and follow-up

This category encompasses self-monitoring with a digital diary, experienced as both a reminder and a motivator, though registering activities could also feel like an extra burden. Different forms of follow-up were described as influencing motivation to remain active over time. Support through multiple contacts with healthcare, both during and after the PAP intervention, was perceived as facilitating sustained activity. Wearing the accelerometer waistband was experienced as increasing awareness of the need to be active and as a motivating factor.

#### Self-monitoring with digital diary as reminder and motivator

Some participants described using the app to register their activity continuously throughout the study, while others described never getting started or stopping after a while. Among those who had used it initially but later stopped, a common reasoning was that registration was no longer needed once the activity had become a habit. Others experienced registering activity in the app as burdensome, particularly when the agreed activity goals were not reached. Participants expressed a wish to be able to comment more freely in the app, to adjust the activity goals more flexibly to better match their current activity level, and to follow their own progress and statistics more closely.

It was fun in the beginning. It was kind of cool that I was in it [the app], that I had a schedule and stuff for what I had done. (C10)

Like as if it was more real, kind of? (Interviewer)

Yes. (C10)

And then that changed and you got tired of it? (Interviewer)

Yes. (C10)

Regular notifications reminding users to register their activity were described as a helpful reminder to stay active. The act of registering an activity, could itself also serve as a reminder to increase activity after a period of inactivity. At the same time, one child experienced the frequent notifications as disruptive, resulting in the parent taking over responsibility for recording.

It was a good reminder. And then you can see for example that there wasn't any activity that week, and then you might push for an extra walk the week after to help up the results.//…// It's a good way to keep [the app] running. So you remember to fill it out and also to push for and support the goals that you set. (P6)

##### Support through multiple healthcare contacts

Regular healthcare contact was described as a facilitating and motivating factor, and some participants enjoyed being able to monitor their results across multiple follow-ups. Being followed over a longer period of time was considered important, both through the PAP treatment and through the study-related visits, which were described as functioning as follow-up in practice. However, others recalled no formal follow-up or described long periods of relative silence from healthcare. More frequent contact, even simple check-ins, was perceived to have the potential to increase motivation, and a desire for more encouragement was expressed.

We had quite a few follow-ups. We came for height and weight measurements I think. And then we talked a bit about how it goes. You also got to ride the bike.//…// I thought it [the contact] was really good actually. To get like a check-up on how it goes and stuff, and get some feedback. It was good to feel that you had some support in that. (P8)

Well, at times it was completely quiet you know. Sure, we can do it on our own, but still—something. Some kind of contact. A chat message would have been fine, just to write something you know. (P5)

#### Wearing the waistband increases awareness

For many participants, wearing an accelerometer waistband on several occasions was a central part of the experience, despite pertaining to the study rather than the PAP treatment itself. Some children found the waistband uncomfortable, particularly around friends at school, though strategies for overcoming this feeling was described and some found it less bothersome than expected. Wearing the waistband increased awareness of physical activity and was described as a reminder and motivator, knowing that their movement was being recorded. There was a wish to be able to access their measurement results, both to compare across timepoints and to receive direct feedback on whether their activity level was sufficient.

I didn’t really have any thoughts, it was more like once you got going, and got those waistbands… how little I move when she's [the child] not home. And also how much you move during a workday. And how little it was after I went on sick leave. How little you move. And… it was like a carrot when you knew that you were going to wear the waistbands. You're gonna get measured. So instead of like sitting around waiting for her when she had practice, well than I would take a walk. (P3)

### Changes beyond physical activity

This category captures experiences and perceptions of changes following the PAP intervention, including changes in the child's physical activity through the adoption of new behaviours and habits, as well as changes in both physical and psychological wellbeing and new insights regarding movement habits and personal development.

#### New behaviours and habits increase physical activity

Increased physical activity was described in different ways; adoption of regular exercise, more active daily life, a change to more active transportation, and increased physical activity also among parents. A few participants, however, described already being active before the PAP treatment and therefore did not perceive a change in their activity level.

Only this little thing… to take a walk. We take the stairs instead of the elevator. Or we go for a swim in the summer. We go bike riding together. If we do something at school, we can take our bikes instead of the car. So it's a bit of a… it makes you think, you know. (P3)

#### Changes in both physical and psychological wellbeing

Beyond physical activity, changes acknowledged by children or parentsincluded improved sleep and reduced weight. Children expressed satisfaction with having participated in the PAP intervention and a general sense of being healthier, which was both recognised by the child itself and acknowledged by the parent. The feeling of being healthier was associated with being more active, for example when riding a bike.

What do you think was the best part of being in the program? About this treatment or this thing about physical activity on prescription? (Interviewer)

That I've become more physical//…// You feel better then. (C5)

You feel that? (Interviewer)

Yes. I feel that I'm better now, healthier. (C5)

On a psychological level, participants described feeling more comfortable in themselves, increased curiosity for activities, and a more daring attitude towards trying new activities.

But you've always liked coming here [to the paediatric clinic]. (P10).

Yes, If I hadn't come here, I don't think things would have turned out the way they are now. I don't think it would have happened at all. (C10)

It would have been different in your life? (Interviewer)

Yes. I'm very glad I participated. My body feels good now. It might not have ended so well otherwise. I wouldn't have lost weight. It's not good to be too overweight. It has helped me. And then I feel more comfortable with myself. (C10)

Yes, you have a completely different self-confidence. (P10)

Yes. (C10)

#### New insights and development

Participation in the PAP intervention was perceived by several participants to have increased their knowledge about physical activity and provided new insights. Participation was described as an eye-opener and a wake-up call towards a more active lifestyle. Being able to follow the child's development in this direction was described as enjoyable.

It's like I said earlier, you've really sort of woken up about this. Like we thought about it the whole time. Or you're aware of it. Uhm… that it's important. To move, you know.//…// So this was the wake-up call we needed so to say.//…// I think it's super hard to say what the study itself did, you know. Uhm… More than making us wake up a little [laughs]. Uhm… Which is a lot in itself. But… it's hard to develop that. (P5)

## Discussion

The main findings of this study were that participating children and parents experienced PAP as an intervention that extended across healthcare, everyday life, and organised sports; that the intervention was perceived as involving both conversational support (“just talk”) and personal responsibility for performing and maintaining activities; and that choosing, initiating, and sustaining activities required consideration of the child's preferences and adaptation to family-related and practical circumstances. PAP was also described as leading to perceived changes in physical activity, along with broader developments in the children's behaviours, physical health, and psychological and emotional well-being. Both children and parents expressed generally positive attitudes towards PAP, and the participants underscored the importance of selecting activities that were both fun and appropriate for the child. Most barriers to participating in physical activities were related to practical considerations such as season or weather, activity costs, and finding time to sustain activities. Key facilitators identified were related to involvement of other family members in the child's activities, selecting fun and child-friendly activities, and maintaining regular activity routines or following structured weekly schedules. Together, the findings suggest that PAP can support families in identifying and integrating suitable physical activities into everyday life.

The overarching theme resonates with socio-ecological theories of behaviour and development, which hold that behaviour change is shaped by interacting influences across multiple levels, with individuals embedded in nested layers of context ranging from the immediate family and social settings to broader institutional and cultural environments (34, 35). This pattern was evident in our data, where PAP was experienced as operating across multiple levels simultaneously. *The clinical encounter* reflects the role of healthcare structures in initiating and framing engagement, while *Finding “your thing”* captures individual-level processes related to preferences and motivation. *Fitting activities into everyday life* illustrates how interpersonal and environmental contexts, such as family involvement and daily routines, shaped adoption and integration of activities. *Motivation through monitoring and follow-up* demonstrates how ongoing support mechanisms, including healthcare contacts and self-monitoring, contributed to sustaining engagement and linked healthcare with everyday life. Finally, *Changes beyond physical activity* suggests that the effects of PAP extended beyond behaviour to broader wellbeing. Together, the findings illustrate how engagement in PAP is shaped across interacting contexts rather than within a single setting.

Our findings are generally consistent with previous research on children's experiences of physical activity promotion and interventions. The findings that physical activities for children need to be fun, socially stimulating, and performed with or supported by friends and family, are supported by both a recent study on PAP in a Swedish school context (36) and by a study that explored experiences of children with cerebral palsy of participation in physical activity (37). In both those studies, children expressed similar preferences for activities that were fun, socially engaging, and supported by family members, underscoring the importance of providing meaningful, manageable, and tailored physical activities in supportive social environments to encourage participation. Similarly, a recent review of qualitative studies examining obese children's and their parents' preferences for physical activities identified several factors influencing motivation and engagement that are consistent with our findings (38). Children expressed preferences for physical activities that were social, fun, informal and regular, as well as the need for whole-family involvement and supportive guidance from healthcare professionals. Barriers identified both in our study and in the review include competing priorities, weather-related constraints, intervention content, and activity-related costs. These findings underscore the need to offer activities that are enjoyable, tailored, socially engaging, and feasible for families.

The generally positive experiences of participating in PAP are consistent with findings from a school-based health promotion programme, in which six-year-old children across weight categories emphasised that of the physical activities tried within the programme the most engaging were the ones that were fun and easy to perform (39). In contrast, older Norwegian children and adolescents reported more negative experiences from participation in a structured group-based lifestyle intervention, describing feelings of pressure, discomfort, frustration, and emotional resistance (40). Although this differs from the largely positive experiences in our study, it reinforces the importance of selecting appropriate activities, ensuring a supportive environment when trying a new activity, and involving the child in decisions throughout the intervention process.

From the parental perspective, studies have highlighted the importance of accessible activities and competent adults who can encourage independence while ensuring safety. One study reported that parents of children with cerebral palsy desire competent support for their child's physical activity and for building friendships, and that family culture and attitudes should be considered when designing interventions (41). Positive experiences were also described by parents of children with obesity of participating in an intervention that combined group sessions with a web-based support programme (26). In that study, parents described the intervention as supportive in promoting healthier lifestyles habits within the family. Participation was perceived as influencing not only the child's behaviour but also the lifestyle of the whole family. However, they also reported challenges related to managing their child's obesity and expressed feelings of parental guilt. These findings resonate with our results, where parents describe both supportive aspects of the intervention and challenges related to sustaining lifestyle changes.

From the healthcare perspective, earlier research has shown that paediatric healthcare professionals generally perceived PAP as an acceptable, appropriate, and feasibly intervention för children with obesity (29). However, several barriers to implementation were identified including lack of activities to suggest, limited time, insufficient organisational support, and a lack of research on PAP for children. While healthcare professionals highlighted organisational and structural prerequisites for implementing PAP, the participants in the present study focused on factors related to the child's motivation and feasibility of activities within the family context.

Taken together, our findings suggest that PAP for children with obesity should integrate child-centred and family-centred components, emphasising fun activities tailored to individual and contextual needs within the child's and family's everyday life. The importance of involving the child in all steps of the intervention, including the decision to participate, cannot be overstated and is essential for motivation, adherence, and sustained engagement. Support for reducing financial and logistical barriers may be crucial for equitable participation. PAP may also benefit from implementation strategies that strengthen cross-sector collaboration between healthcare, schools, and community sports and activity organisations. Future research should evaluate the feasibility and effectiveness of PAP in paediatric populations at scale, examine long-term behavioural and health outcomes, and further explore how socio-cultural factors influence engagement among diverse families.

### Methodological considerations

Interviewing children comes with unique challenges related to the child's cognitive and social development, requiring careful consideration in the interview situation. Examples of such challenges were restlessness, limited attention span, and an expressed desire to finish the interview, which all may have influenced the child's answers to interview questions. Aware of these challenges, the interviewer was prepared with visual aids and paper and crayons, which were used at times to redirect the child's attention and at other times to maintain engagement, for example when talking about activities. Another challenge was the ability of the children to recall the different events included in the PAP intervention. Interviews were conducted as soon as possible after the 12-month follow-up, to capture experiences of both the intervention and the subsequent follow-up period. Although kept to a minimum, the time elapsed from the intervention start and the initial PAP consultation to the interview may have introduced recall bias, particularly regarding the early intervention components counselling and prescription. Earlier research has suggested that children's accounts in interviews might be influenced by memory bias, and that children's memory for conversational statements (such as the initial PAP counselling session) may be sparse and unreliable (42).

Although the number of participating families was limited, interviewing child–parent dyads enabled triangulation and comparison of complementary perspectives on the same intervention experience, which contributed to interpretive richness and the development of categories and theme. To help determine whether the sample size was sufficient, we assessed information power. This concept reflects the adequacy of a sample in relation to the study's aim, sample specificity, use of theory, quality of dialogue, and analytic strategy rather than a fixed numerical threshold (31). As no substantively new information emerged in the last interviews [often referred to as data saturation or informational redundancy (43)], the material was deemed sufficiently rich to address the research question and the sample size of 22 was considered adequate*.*

Interviewing the child and their parent together also entails challenges related to the child's integrity. The presence of the parent may have influenced the child's sense of independence and their perceived ability to speak freely. While supportive parents can encourage openness on the part of the child by contribution to a safe environment and by providing more factual information, disapproving parents could have the opposite effect (44). The decision to conduct joint interviews in this study was deliberate. This approach was chosen primarily to provide a sense of security for the child and to ensure a supportive environment, with particular consideration of the stigma this vulnerable patient group often suffers from. It also enabled the identification of potential agreements and disagreements between the child and the parent.

A further methodological challenge was distinguishing participants' experiences of the PAP treatment and outcomes from those related to the study procedures. To facilitate this distinction, a visual aid describing the different components was used in the interviews. Nevertheless, for many participants—particularly the children—the study components, such as assessment appointments, wearing the accelerometer waistband, and contact with research staff, were perceived as integral parts of the PAP experience. As these aspects appeared to influence participants' activity behaviour, we chose to include them in the analysis. This conflation of intervention and study components poses a potential threat to construct validity and should be kept in mind when interpreting the findings. At the same time, the motivating role attributed to wearing the waistband and being monitored underscores the value of systematic follow-up and self-monitoring as part of PAP.

Given the predominantly positive accounts, negative experiences may potentially be underrepresented. This is particularly relevant in the context of childhood obesity, where feelings of stigma, discomfort, or resistance to intervention may be difficult to articulate—or to share with an interviewer. Selection bias and social desirability bias may have contributed to this underrepresentation; participants with positive experiences probably were more likely to participate, and children in particular may have provided answers that they believed were expected or desired from them. Interviews being performed in the presence of siblings may also have affected both children's and parents' responses, with a possible overweight of positive statements not to disappoint one another.

Trustworthiness was considered throughout the research process, with attention to the coherence of a “red thread” from the study aim and methodological choices to the analysis and presentation of findings (33). Credibility was addressed through the inclusion of participants with relevant experience of the phenomenon and through data that allowed for variation in the content. The inclusion of both children and parents enabled multiple perspectives in data collection. A challenge in the data collection was capturing the perspectives of the youngest participating children, as they were generally less talkative than both their older peers and their parents. Having a parent present in the interview can influence the interview process positively or negatively, e.g., by creating a safe environment for the child or by hampering the child's own narrative, respectively (44). Despite consistent efforts in the interviews to always address the child first, the parent would sometimes act as a proxy for their child. Similarly, the non-native children were also less talkative, implying linguistic limitations that may have hampered the depth of these interviews. At the same time, our decision to include non-Swedish-speaking families represents an important strength of the study, as it enabled us to identify barriers related to being new in Sweden and unfamiliar with Swedish cultural norms, e.g., with organised sports.

Dependability was strengthened by a systematic and transparent analysis process, including ongoing discussions among the researchers to reflect on coding, categorisation, and the influence of pre-understandings. The two authors responsible for the initial analysis collaborated in all steps and repeatedly discussed and acknowledged their preunderstanding. Using an additional analyst to verify the analysis further strengthened dependability. Confirmability and authenticity were supported by grounding interpretations in the data and by presenting representative quotations, thereby giving priority to the participants' voices.

To facilitate transferability, the study context and participants were described in sufficient detail, allowing readers to judge the applicability of the findings to other settings (33). Transferability was also enhanced by the inclusion of families of non-Swedish origin, reflecting the current population mix in Sweden and potentially enabling the transfer of study findings to other contexts. However, a potential limitation to transferability could be the participants’ difficulty in distinguishing intervention components from study components. This may have rendered their experiences broader than just the PAP treatment, limiting the possibility to transfer the findings to individuals who only participate in an intervention. Lastly, the skewed sex distribution of participating children was an unintentional consequence of the limited number of eligible participants from the underlying study and may potentially reduce transferability further.

## Conclusions

This qualitative interview study shows that children with obesity and their parents experienced PAP as a supportive, person-centred intervention that extended beyond healthcare into everyday life and organised sports. The findings suggest that PAP is perceived as combining conversational guidance with the child's and family's own responsibility to initiate and maintain activities. Engagement can be facilitated when activities are fun, tailored to the child's preferences, and compatible with family routines, while costs and practical challenges can be substantial barriers for participation. Physical activity on prescription may also yield broader positive effects on the child's behaviours, physical health, and psychological well-being. These findings highlight the importance of individually adapted, family-inclusive approaches and suggest that PAP may serve as a feasible and acceptable strategy for promoting physical activity in paediatric obesity care.

The study contributes to a better understanding of PAP as a treatment method for children with obesity and the feasibility of implementing it in Swedish paediatric healthcare. The increased knowledge about how children and parents experience participating in PAP is important for future work with adapting and implementing the method for this population, as well as for future research on the use of PAP in paediatric healthcare.

## Data Availability

The datasets presented in this article are not readily available because access to the data supporting the findings of this study is restricted in accordance with ethical approval conditions and data protection requirements. Requests to access the datasets should be directed to the principal investigator at susanne.bernhardsson@vgregion.se.
