# Supplementary figures and images for: Experiences of children with obesity and their parents of participating in a physical activity on prescription intervention: a qualitative study
Source: Front Pediatr. 2026 May 14;14:1831386. doi: 10.3389/fped.2026.1831386 (PMC13215984; doi:10.3389/fped.2026.1831386)

**Supplemental file 1 Visual aids**


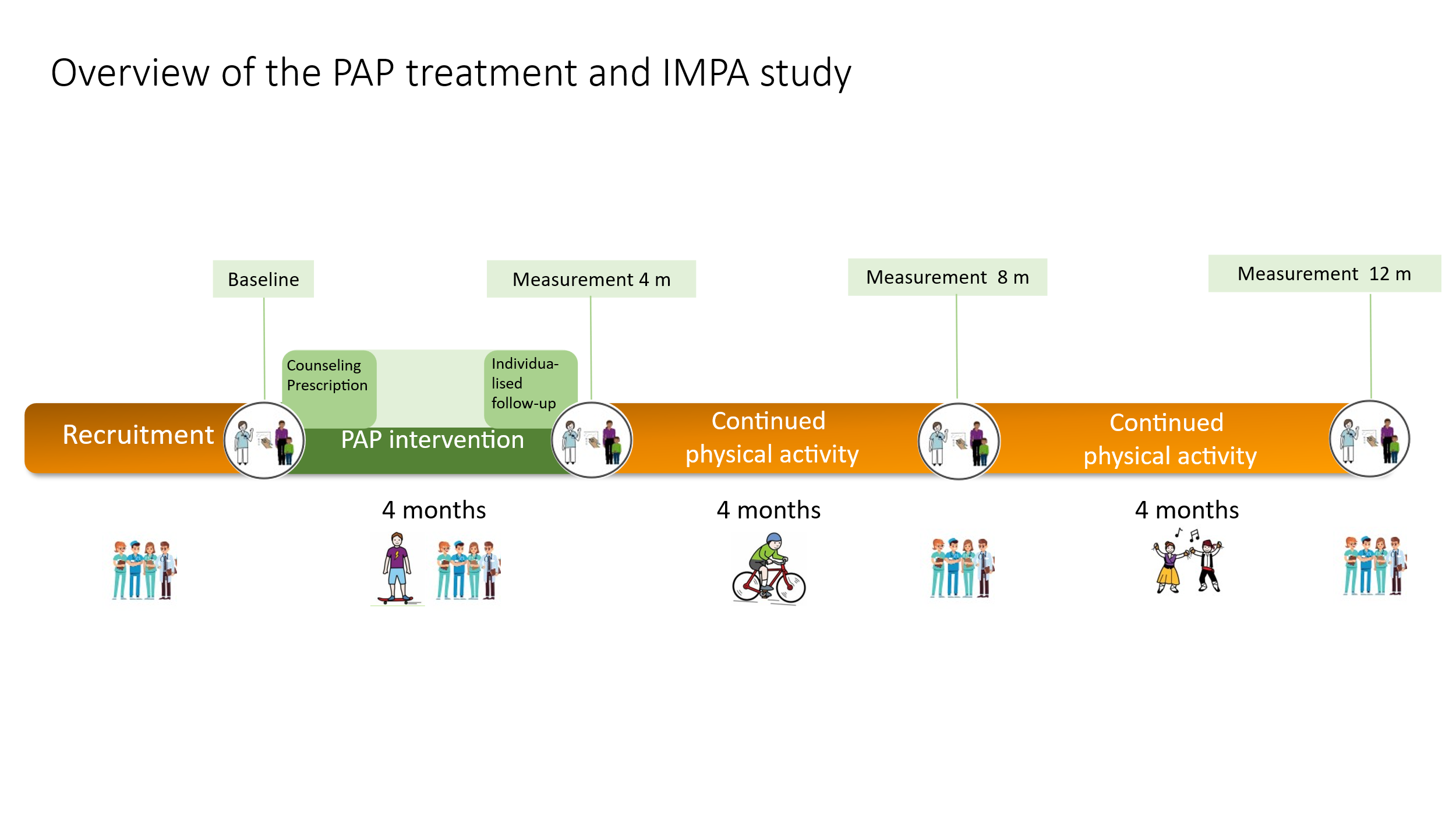


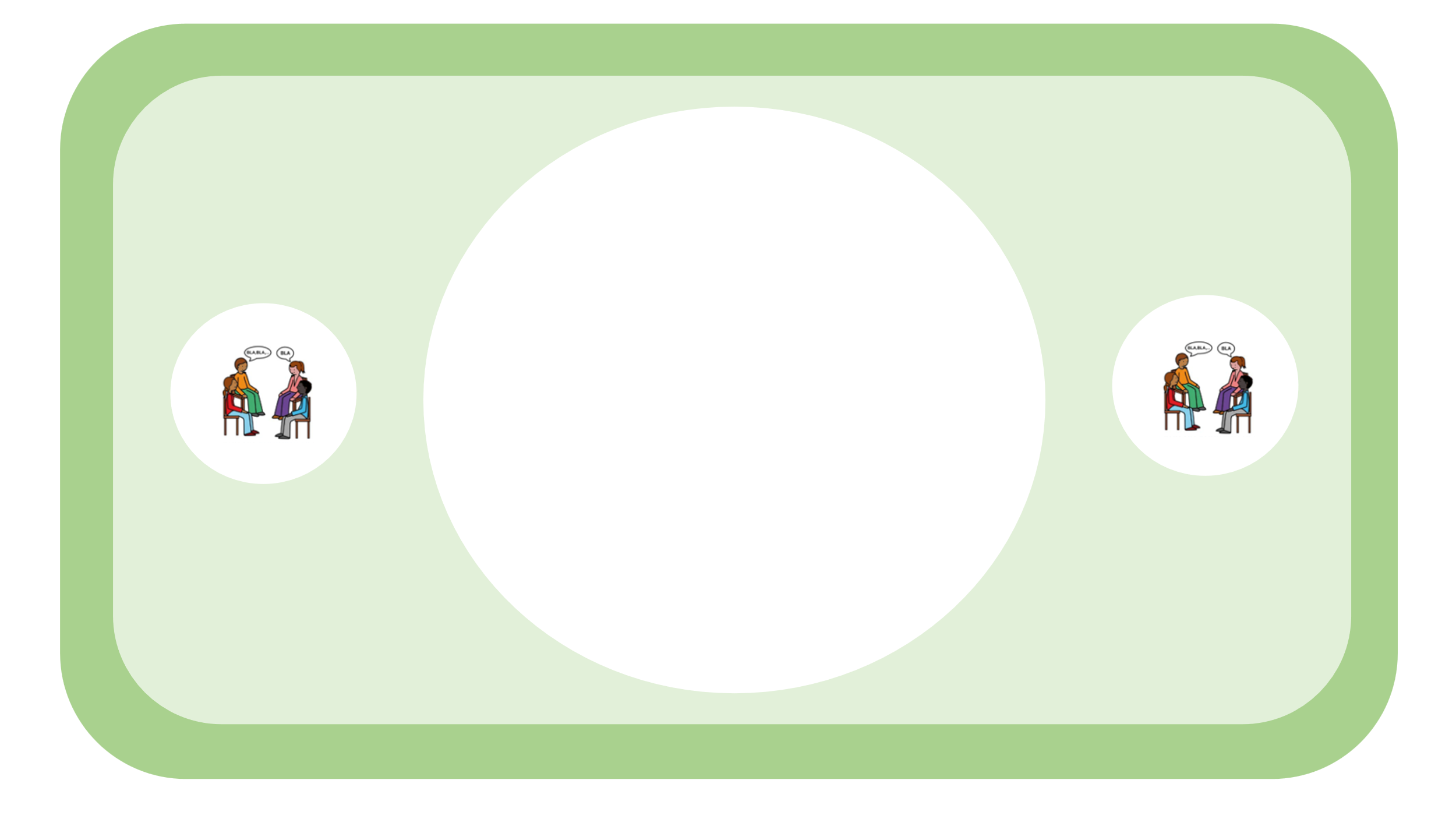


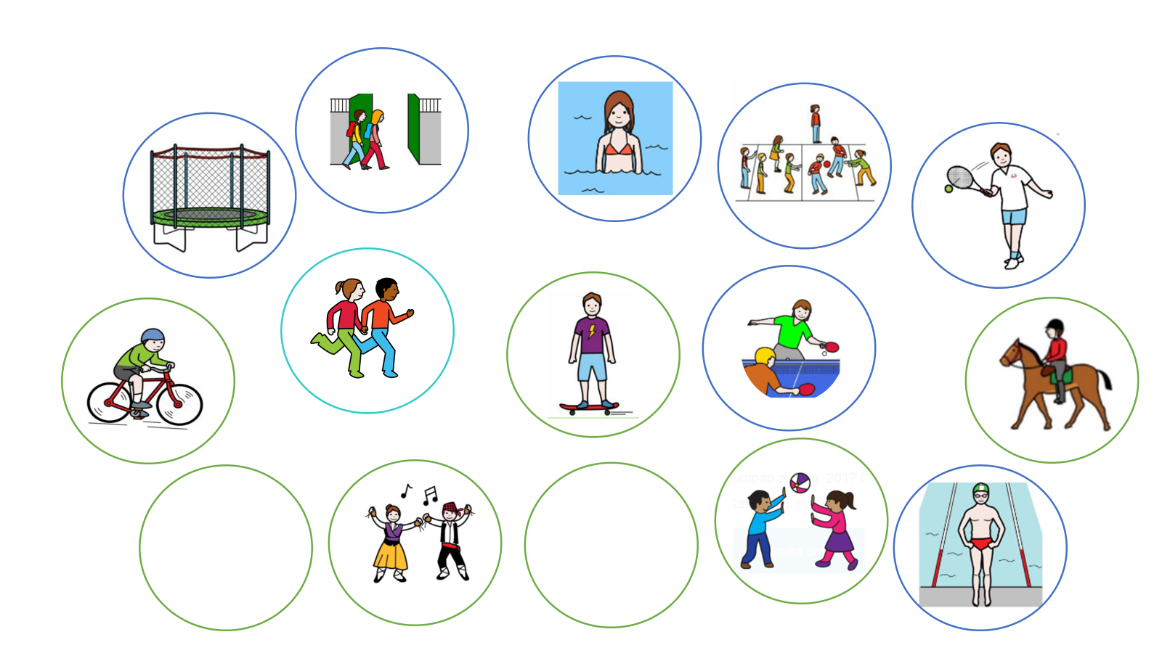

Supplement: Supplementary file 1 [file Supplementaryfile1.docx]
