# Supplementary material for: Experiences of children with obesity and their parents of participating in a physical activity on prescription intervention: a qualitative study
Source: Front Pediatr. 2026 May 14;14:1831386. doi: 10.3389/fped.2026.1831386 (PMC13215984; doi:10.3389/fped.2026.1831386)
Supplement: Supplementary file 2 [file Supplementaryfile2.docx]

**Interview guide**

**Introductory question**

You have just finished your participation in the IMPA study, where you received a treatment called Physical activity on prescription) (in Swedisg FaR – Fysisk aktivitet på recept).

***Can you tell me what it was like to participate in this treatment? What was the treatment about for you?***

**Key questions (if they don’t come up already in the introductory question)**

***Can you tell med about the visit to the nurse in the beginning of the treatment where you taled about different activities?***

***Please tell me about the prescription you received.***

***What was it like to do the activities?***

***Please tell me about the contact you had with the nurse during the 4 months of the treatment?***

***What has your physical activity been like after the treatment? Is there any difference in how active you are? More, less, or the same; how/which type of activity?***

***What was the best part of the treatment?* *And the worst?***

**Summing up (reflection over what was said)**

***Of everything we’ve talked about today concerning your PAP treatment, what do you think is the most important for us to know?***

***Is there anything else you’ve thought about that we haven’t talked about today?***

***Is there anything that’s unclear, do you have any questions?***

**General prompts/follow-up questions to clarify and expand the answers**

*-Can you tell me more about…?*

*-Can you explain some more….?*

*-Can you give an example?*

*-What did you think then?*

*-How did you feel then?*

*-Do you have other examples?*

*-How do you mean?*

*-The thing you said about… was interesting, can you tell me more about that?*
